# Exercise-Based Muscle Development Programmes and Their Effectiveness in the Functional Recovery of Rotator Cuff Tendinopathy: A Systematic Review

**DOI:** 10.3390/diagnostics11030529

**Published:** 2021-03-16

**Authors:** Juan G. Dominguez-Romero, José J. Jiménez-Rejano, Carmen Ridao-Fernández, Gema Chamorro-Moriana

**Affiliations:** Research Group “Area of Physiotherapy” CTS-305, University of Seville, 41009 Seville, Spain; j_gab_dom@hotmail.com (J.G.D.-R.); jjjimenez@us.es (J.J.J.-R.); mcrf.2817@gmail.com (C.R.-F.)

**Keywords:** physiotherapy, rotator cuff, tendinopathy, exercise, resistance training

## Abstract

(1) Background: Rotator cuff (RC) tendinopathy causes pain and functional limitation of the shoulder. Physical exercises are effective therapies but there is no consensus on which exercise programme is the most appropriate. Objective: To analyze and compare the effectiveness of different intervention modalities-based exclusively on physical exercise muscle-development programs to improve shoulder pain and function in RC tendinopathy. (2) Methods: Systematic review (PRISMA) through a search in PubMed, WOS, PEDro, Cinahl, Scopus and Dialnet. The PEDro Scale and the Cochrane Risk of Bias analyzed the methodological quality. A pre-established table collected data on: patients, interventions, outcome measures and results. A narrative synthesis of the results was conducted. (3) Results: eight articles were selected (Cochrane: *low risk of bias*; PEDro: good quality). All assessed programs were effective. Only one study found statistically and clinically significant differences in favour of eccentric training. The exercises used were: eccentric/concentric/conventional, open/closed kinetic chain, with/without co-activation of glenohumeral muscle, with/without pain, and in clinic/at home. (4) Conclusions: All exercise programs were effective in RC tendinopathy, improving pain and shoulder function. No solid results were obtained when the interventions were compared due to their heterogeneity. Patients perception assessment tools were the most widely used. Amount of load applied should be considered.

## 1. Introduction

Shoulder pain is one of the most common symptoms of musculoskeletal injuries, and a quarter of the general population and up to two-thirds of adults suffer from it at some point in their lives [1]. Its prevalence has been estimated at 15–30% of the population [2]. The most common cause of shoulder pain is rotator cuff (RC) tendinopathy [3]. This structure surrounds the shoulder and is composed of the supraspinatus, infraspinatus, subscapularis, teres minor and long portion of the brachial biceps [4], although some authors do not include the latter [5,6]. In general, tendinopathies are common “overuse” injuries and are characterised by load-related pain and loss of function [7]. A 2019 symposium on tendinopathies [8], considered the terms “subacromial pain syndrome (or impingement)” and “rotator cuff-related shoulder pain” referring to pain in the shoulder tendons with loss of function, to be synonyms. The symptomatology of this pathology includes: minimal pain at rest with a broadly preserved range of motion (ROM) and increased pain in resistance training [1]. Evidence shows that disability caused by RC tendinopathy has a significant impact on daily life and can lead to a social burden due to absenteeism and health resource costs [9]. Half of those affected experience these problems for more than 12 months continuously and often end up undergoing surgery [10].

The tendency is to consider that the aetiopathological mechanism of RC tendinopathy is produced by acute and chronic changes in the tendon structure (increase and changes in collagen, proteoglycans, vascularization and cells), as well as in the surrounding bursa (increase in inflammatory proteins: pain mediators and bursa matrix modifiers) [11]. Despite this, both the aetiology and physiopathology of this tendinopathy, as well as its relationship with the subacromial bursa, should be studied further [11]. It is a multifactorial lesion combining intrinsic, extrinsic and environmental factors [11]. Lewis [12] considered that RC tendinopathy could be adjusted to the continuum model of tendinopathy of Cook et al. [7,13], which sustained this pathology was dynamic and degenerative. It implied three phases: reactive, failed healing (known as the dysrepair phase) and degenerative. All of them could be reversed apart from the degenerative phase. The natural history of this tendinopathy was not always favourable, and in the long term the results of physiotherapy interventions were often poor [1].

RC tendinopathy suffers from pathological changes similar to those of lesions in other tendons, such as the epicondyles [14], Achilles tendon [15] and patellar [16]. In all of these, physical exercise-based therapies have shown their usefulness in achieving functional recovery. In fact, previous studies [17,18,19,20] showed that a programme of physical activity with progressive overload is effective for RC tendinopathy. Different types of physical exercise are used: concentric, eccentric, proprioceptive, high-load, etc. In addition, they could be directed at different structures, such as the cuff or the scapular muscles. Yet there is no consensus on which exercise programme is the most appropriate, since several authors show contradictory results [21,22,23,24]. On the other hand, even if this type of therapy modality (physical exercise) has been proven effective for the RC, numerous systematic reviews [17,25,26,27,28] insist on the need to improve the methodological quality of the studies to reach sound conclusions.

Given the current lack of knowledge as to which exercise programme is more effective in treating RC tendinopathy, knowing that this activity is an effective therapy; its high prevalence; and the fact that this pathology entails heavy spending, either on health care or in work terms; this study aimed to analyse and compare the effectiveness of different intervention modalities-based exclusively on physical exercise muscle-development programmes to improve shoulder function and pain in RC tendinopathy patients.

## 2. Materials and Methods

This systematic review followed the guidance of the PRISMA statement [29]. The protocol was published on PROSPERO with registration number CRD42020220098.

### 2.1. Search Strategy

A search was performed from inception through 31 December 2020 on six electronic databases: PubMed, WOS, PEDro, Cinahl, Scopus and Dialnet. Medical subject headings (MeSH) terms were employed. Other terms of interest were also included due to its frequency in scientific studies. “Search strategy terms ordered by meaning” and “Search strategy in the different databases” are shown in Table A1 and Table A2 (Appendix A and Appendix B), respectively.

### 2.2. Study Selection and Inclusion Criteria

- Participants: Adults (≥18 years) with signs and symptoms compatible with RC tendinopathy in the absence of other shoulder diagnoses (ruptures/tears RC, frozen shoulder, shoulder instability).
- Intervention/comparison: Studies that include and compare programmes of active physical exercise involving gravity-resistance, either actively and freely or with an extra load applied (dumbbells, elastic bands, pulleys, etc.). Programmes consisting exclusively of active exercises (excluding manual therapy, electrotherapy, pharmacology, etc.) were allowed. Two exceptions are considered: passive stretching, as we understand that they usually form part of these programmes for final relaxation purposes; and the use of corticosteroids up to 2 months before the intervention, since most studies allowed this.
- Measurements: Studies that include measurements of shoulder strength and ROM, as well as of patients’ perception, i.e., “Patient Reported Outcome Measures” (PROMs), of pain and function by means of functional assessment scales.
- Study design: Randomised Clinical Trials (RCTs) with a minimum of 6 points on the PEDro scale [30].
- Language: English.

### 2.3. Data Extraction

Data extraction was carried out by one reviewer (JD) and checked for accuracy by a second reviewer (CR). Disagreements were resolved by a third reviewer (GC). A pre-established table was used to detail the information on study characteristics: identification, objectives, participant characteristics (gender, age and inclusion/exclusion criteria), interventions, outcome measures and results of the selected studies.

### 2.4. Methodological Quality Analysis

Included studies were assessed for methodological quality using Physiotherapy Evidence Database (PEDro) critical appraisal tool. This method is valid and reliable for evaluating the internal validity of the studies [30]. PEDro consists of 11 items, although criterion 1 refers to the external validity of the paper and is excluded from the final result. Each criterion can be Yes (1 point) or No (0 points), with a maximum punctuation of 10. A total score of 6 or more is considered to be a *good* methodological quality (6–8 good; 9–10 excellent), and a total score of 5 or less is related to a limited methodological quality [31]. This valuation was complemented using Cochrane Risk of Bias Tool (CROB). It consists of six domains with the following score: high risk, low risk and unclear risk [32].

## 3. Results

### 3.1. Literature Search and Selection

**Figure 1:**
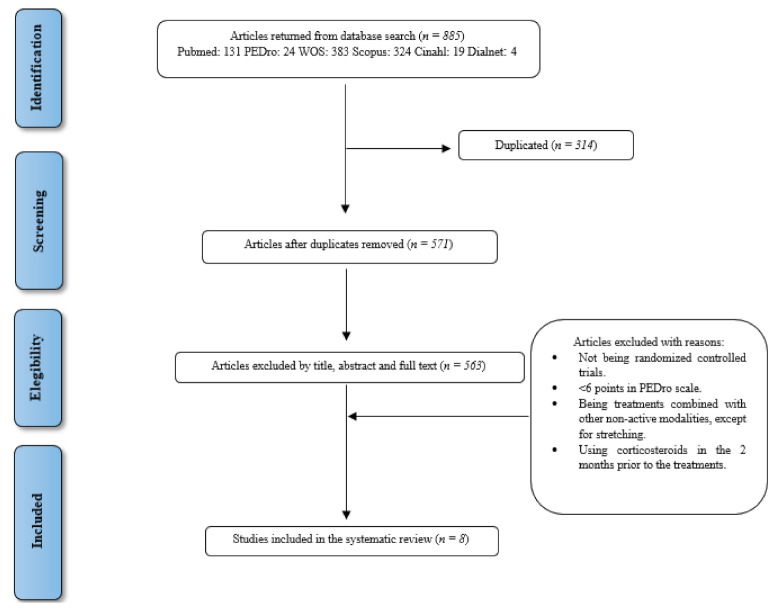
PRISMA flow diagram.

The literature search identified 885 records, of which 314 were duplicates. After screening the titles, abstracts and full text of the remaining 571 studies, 563 papers were excluded, and 8 papers were included in the review. Figure 1 shows the search and study selection process following PRISMA statement [29].

### 3.2. Characteristics of Included Studies

A detailed summary of the characteristics of each study can be found in Table 1.

### 3.3. Assessment of Methodological Quality

The level of evidence of this systematic review is 1b according to the CEBM Classification (Centre for Evidence-Based Medicine; Oxford) [38].

The results of the PEDro scale are shown in Table 2.

**Figure 2:**
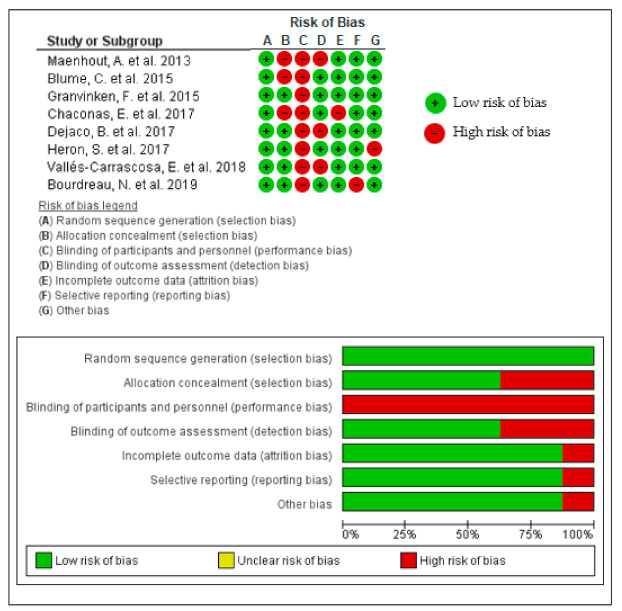
Assessment of the methodological quality of the studies using Cochrane Risk of Bias Tool for Randomised Trials (summary and graph).

The results of the Cochrane Risk of Bias Tool are shown in Figure 2.

In short, regarding PEDro, all studies showed *good* methodological quality (between 6 and 8), with a minimum score of 6 being an inclusion criteria. Despite the good score, it should be noted that none of the studies scored the items: “There was blinding of all subjects” 5 and “There was blinding of all therapists who administered the therapy” 6. Conversely, the items “Subjects were randomly allocated to groups” 2, “Groups were similar at baseline regarding the most important prognostic indicators” 4, “The results of between group-statistical comparisons were reported for at least one key outcome” 1 and “The study provides both points measures and measures of variability for at least one key outcome” 11 were scored by all studies.

Regarding CROB, all studies showed “*low risk of bias*”. The domains “blinding of participants and personnel (performance bias)” was scored by no study and the “random sequence generation (selection bias)” was scored by all studies.

In general, the results obtained using the PEDro (*good)* were consistent with those of the CROB (*low risk of bias*).

### 3.4. Participant Characteristics

A total of 409 adult participants, 198 women and 211 men, were studied. The studies used a sample size between 18 [39] and 120 subjects [23].

As for the inclusion criteria of the papers, five of them [23,24,33,35,36] established a symptom duration of at least 3 months. All the studies, except one [22], used orthopaedic shoulder tests to evaluate participants’ inclusion in the study. Table 3 represents the different assessment tests used in the studies’ inclusion criteria and how many times these tests were employed.

### 3.5. Characteristics of the Interventions

The studies compared different muscle development exercise programmes involving gravity-resistance, either actively and freely or with an extra load applied (dumbbells, elastic bands, pulleys, etc.): concentric vs. eccentric exercises [34]; open kinetic chain vs. closed kinetic chain vs. mobility exercises [23]; exercises with co-activation of glenohumeral muscles vs. without it [37]; exercises with pain vs. without pain [22]; eccentric exercises vs. conventional therapeutic exercises, i.e., a typical exercise programme for RC tendinopathy [24,36]; exercises with high eccentric load vs. without it [33]; supervised exercises in clinic vs. exercises at home [35]. The studies were grouped according to the predominant muscle contraction: concentric, eccentric and both of them (Table 4).

The content of the exercise programmes was heterogeneous, although in general it consisted of exercises with resistance bands, functional exercises (bending, standing up from a chair, etc.) and, despite the exclusivity, as an exception, passive stretching exercises. The interventions lasted from 4 [22] to 12 weeks [24,33]. Only three studies [24,33,36] followed up patients until week 26 (Table 5).

### 3.6. Outcome Measures of the Selected Studies

Dynamometers were used to measure isometric muscular strength of the shoulder in ABD [24,33,34,36], ER [33,34,36] and IR [33,36]. Digital inclinometers and goniometers were employed to measure shoulder ROM in flexion [22,24,35], extension [22], ABD [22,24,34,35], adduction [22], ER [22,24,35] and IR [22,35]. All the selected studies measured the patients’ perception of pain, function, fear, etc., through PROMs (Table 6).

### 3.7. Narrative Synthesis of the Results of the Selected Studies

The results were presented on the basis of a comparison between the different exercise programmes used:

- Concentric vs. eccentric training

Based on one RCT (*n* = 34) [34] with a low risk of bias (PEDro scale), there were no significant differences when using exercise programmes with concentric or eccentric contractions for the improvement of shoulder pain, function, ROM and strength.

- *Open kinetic chain vs. closed kinetic chain vs. mobility exercises*

Based on one RCT (*n* = 120) [23] with low risk of bias (PEDro scale), there were no statistically significant differences between groups for improvement of shoulder pain and function, and no clinically relevant differences were found in the primary variable “Shoulder Pain and Disabilities Index” (SPADI).

- *Exercises with vs. without co-activation of glenohumeral musculature*

Based on one RCT (*n* = 42) [37] with low risk of bias (PEDro scale), there were no statistically significant differences between groups for shoulder pain, function and acromio-humeral distance (AHD).

- *Exercises with pain vs. without pain*

Based on one RCT (*n* = 22) [22] with a low risk of bias (PEDro scale), both exercise methodologies were seen to significantly improve shoulder pain, function and ROM without differences between them.

- *Eccentric training vs. conventional therapeutic exercises*

Based on 2 RCTs (*n* = 36) [24] and (*n* = 48) [36] with low risk of bias (PEDro scale), different results were found. While the first study found that both exercise methodologies improved shoulder pain and function significantly without finding differences between them, the second showed statistically and clinically relevant differences for pain and function using an eccentric exercise programme.

- *Exercises with vs. without high eccentric load*

Based on one RCT (*n* = 61) [33] with a low risk of bias (PEDro scale), it was argued that both methodologies significantly improved pain, function and strength, but no differences were found between them.

- *Supervised exercises vs. exercises at home:*

Based on one RCT (*n* = 46) [35] with low risk of bias (PEDro scale), there were no statistically significant differences between the two methodologies for the improvement of pain and function. No participant reported full recovery after treatment.

To summarize, the narrative synthesis showed, in relation to the effectiveness of the interventions, that only one study [36] found statistical and clinical significance between the groups (Table 7).

## 4. Discussion

This systematic review analysed and compared the efficacy of different intervention modalities based exclusively on muscle-development physical exercise programmes for the improvement of shoulder pain and function in patients suffering from RC tendinopathy. Based on the results obtained, all therapeutic modalities of physical exercise improved both variables in a similar way, without highlighting the effectiveness of one over the other.

Physiotherapists, especially in the field of traumatology, usually recommend some kind of physical exercise for RC tendinopathy [40]. These include scapular proprioceptive exercises and those specific to the RC [40]. In a survey of 502 physiotherapists [40], the most commonly used type of exercise was isometric (60.2%). Isometric exercises proposed by Rio et al. [41,42] for patellar tendinopathy were extrapolated to the other tendons [43]. This type of muscle contraction was considered by none of the studies included despite its relevance.

Exercise in general is an effective therapy for shoulder pain [44] and particularly for RC tendinopathy [17], as stated in the introduction. According to the continuum model of tendinopathy of Cook et al. [7,13], exercise is the key treatment for this pathology because it produces cellular and structural changes [45,46,47,48,49]. However, it is not known which muscle development-based exercise programme is best for RC tendinopathy due to the wide variety of exercise methods used at present based on this review. Furthermore, these results are in line with those presented in the systematic review of Littlewood et al. in 2015 [44], where there was no scientific evidence on the most appropriate parameters to apply in active exercises. This, in turn, implied that the studies used different types and parameters of exercises, making it difficult or impossible to compare their efficacy and to carry out meta-analyses.

Passive stretching is quite often included in muscle-development exercise programmes as a final relaxation, so it was allowed in this review-based exclusively on muscle-development physical exercise programmes. This type of stretching is effective in pathologies such as osteoarthritis [50] or rheumatoid arthritis [51]. However, when it is combined with other therapies such as muscle development or balance, together with the methodological limitations of some studies [52,53], their usefulness in active exercise programmes could be questioned.

There are three areas in terms of how the tendons adapt to load [54]: the absence of load area and the excessive load area, resulting in a maladaptive response of the tendon with increased degradation of collagen, as well as a third adequate load area, where homeostasis and functional adaptation of the tendon are maintained. Again, based on the model proposed by Cook et al. [7,13], and taking into account the load progression model for Achilles and patellar tendinopathy [55], it is established that each patient has a specific load tolerance evaluated with a specific load test. This particularity enabled the thought that more scientific evidence should be generated on how the appropriate load is assessed and managed, rather than looking for a specific exercise. Exercise will be beneficial or not depending on how hard the patient finds it, and could be one of the reasons why no differences are found when comparing several types of physical exercise.

Following the background, different muscle-development exercises could be used for not establishing the adequate therapeutic objectives according to each aetiopathology, among other reasons [56]. For example, some studies used the term “subacromial syndrome”, as they understood the “subacromial impact” to be the cause of the clinical problem. However, overuse is actually considered the cause of the tendinopathy [57]. Therefore, this term should not be used to avoid confusion and to agree on a common language [57]. This fact leads to the criteria used by researchers for inclusion of study subjects. In this regard, it should be noted that the articles analysed in this review considered similar inclusion criteria. However, the majority (87.5%) used orthopaedic tests, such as the Neer or Hawkins-Kennedy test, which show poor specificity and lack of diagnostic validity for subacromial impingement syndrome [58,59]. These tests aim to isolate a specific structure of the shoulder. For example, the *empty can test* and the *full can test* [60] focus their attention on the supraspinatus tendon and muscle [61]. In contrast, anatomical and histological dissections show the interlocking nature of the RC tendons and their relationship with the capsule, ligament and bursa [62]. Furthermore, this argument is supported by the fact that, during the *empty can test* and the *full can test*, the supraspinatus is not specifically activated, but when these tests are being performed, up to nine and eight muscles, respectively, are activated [63]. Therefore, it should not be expected that a specific structure is isolated by this type of test. Simplifying shoulder pain to a structure is a very reductionist approach that does not take into account other factors that may influence it, for example, depression, anxiety or insomnia [64], because no nociception is needed to generate pain [61].

Regarding methodology, a large part of the papers that analyse exercise combine it with other therapies [65,66,67,68], which makes it difficult to know the real effect of exercise. Thus, other therapies were excluded in this review. Additionally, these were studies of poor methodological quality [69,70,71], so no solid conclusions can be drawn. To verify this last point, the use of methodological quality tools from the selected studies is highly recommended [30,32]. According to recent studies [72,73], PEDro and CROB are considered valid and useful methods for assessing RCTs. PEDro covers more items than CROB, although six of them are common, which makes the content similar [72]. Despite the aim of both of them being common and their content similar, they should not be used interchangeably due to the low convergent validity of their summary scores and of some individual items [72].

Furthermore, there is currently no consensus about the most appropriate choice between the methods mentioned [73], so more research is necessary to evaluate the best option for each case. Regarding physiotherapeutic interventions, as is the case at hand, PEDro (1999) was designed and recommended especially for them [74,75], although there are authors who advise against it, as it provides a global score that aggregates heterogeneous items, which can mask interest biases [76,77]. By contrast, CROB, created in 2011 [73], evaluates fewer domains, but does so in an isolated way [32,78]. In addition, despite it also offering a global interpretation, it is not based on aggregating points, but taking into account whether the biases produced, i.e., items negatively scored could have a significant impact on the results or conclusions of the study [78], i.e., risk of material bias. Thus, CROB involves a clear subjectivity component regarding the reviewer, which differentiates it from PEDro, and which has led researchers, among other factors, to a lack of consensus regarding best method.

The authors of this study, after evaluating expert opinions, decided to include PEDro and CROB, not only to evaluate the possible biases of the selected studies, but to compare the global interpretation of the results of both of them: *good* (PEDro) and *low risk of bias* (CROB). Although these global data were consistent among them, some items need to be discussed individually. In this sense, it is interesting that the intrinsic nature of physiotherapy interventions often makes it difficult to blind both patients and the physiotherapists assisting them. This means that scoring of clinical trials using scales of methodological quality that consider blinding, such as the PEDro and the CROB, has this initial handicap, as opposed to other clinical settings. That could be why none of the RCTs analysed used a placebo group to assess the impact of the intervention [79,80]. This fact became clear in our results. Although the three items related to blinding in PEDro are objective and easy to evaluate (Yes/No), the two in CROB led us to wonder if their non-compliance would imply a global interpretation of “*unclear risk of bias*” and not the established one, “*low risk of bias*”. On the one hand, this reflection becomes more important when knowing that one of the variables considered was pain and that patients blinding in these cases is considered essential [81,82]. However, the interventions considered in this study were based on physical exercises, without the use of placebo control groups in any case. Both groups did exercises that were beneficial for shoulder pain and function, as the results showed. This fact made user blinding, and even that of the professional, less important. Therefore, we consider that the conclusions would not have been different with double blinding. Regarding the “blinding of outcome assessment”, which three articles did not comply with, we observed that most of the results were objective, quantifiable and therefore not modifiable by the evaluator. For example, PROMs, goniometers, inclinometers and digital dynamometers are assessment tools that provide numerical results. Few authors used manual goniometers, which imply a certain subjectivity, but, when used correctly by experts, minimise bias [83,84]. In addition to the reasons mentioned, [32] states that when blinding is not feasible in a trial, its quality should not be considered low.

On the other hand, we valued the “random sequence generation” item as very important in these interventions, which was met by all studies, as it was one of the inclusion criteria for the systematic review.

To conclude the discussion on the assessment of methodological quality, based on the justifications stated above, the authors decided on the “*low risk of bias*” interpretation of the CROB, but with some caution due to its tendency towards “*unclear risk of bias*”, unlike the clear “*good*” result obtained with PEDro. Likewise, this review advocates presenting the global results of any assessment of methodological quality, and the individual data of each item, as recommended by [32], as well as a summary that describes the most notable aspects and even a justification of the subjective substantial decisions, which, as is the case for the CROB, need to be made.

The studied interventions lasted from 4 to 12 weeks. In this regard, there is evidence of an improvement in strength and in the area of the muscular cross section in a period of 2 to 4 weeks [85,86]. Authors such as Fridén [87] have established that, specifically, eccentric training requires 8 weeks to generate structural alterations in the skeletal muscle. Therefore, the question is whether a period of 4 weeks of training is enough to generate useful changes in the recovery process as proposed by Vallés-Carrascosa et al. [22].

Regarding the outcome, the measures used most often to assess patient perception in this review are PROMs. These questionnaires, with subjective connotations, are increasingly frequent in the scientific literature on shoulder studies [88]. In this regard, Mosher et al. [88] established that the most widely used were the *“American Shoulder and Elbow Surgeons Shoulder Score”, “Oxford Shoulder Score”* and *“Visual Analogue Scales”*. However, the tools considered most often in this research were NPRS or VAS for pain, and SPADI and WORC for function and pain. Communication during a therapeutic process between clinicians and patients tends to focus on the latter. Therefore, it not only encompasses objectivity, but also involves users’ feelings, ideas, concerns and experience about their health condition, i.e., more subjective aspects [89]. Clinicians should know both the patients and their context to generate a therapeutic alliance and, consequently, improve treatment adherence, even more so if it is active physical exercise [90]. Using these measurement systems encourages patients to participate more actively in their treatment through self-assessment of pain [91], function [92] or fear [93], among others.

One of the strengths of this study is that it involved an extensive literature search in six databases with no time limit. RCTs were only included to achieve the highest level of evidence possible in clinical trials, as well as to ensure a low risk of bias in the methodology. All studies were scored on the PEDro scale of *good* methodological quality, and a minimum of 6 points was required in the inclusion criteria. This review presents a qualitative analysis of active exercise programmes that increase muscle tone in order to improve pain and function in the shoulders with RC tendinopathy. Secondarily, the approach of the studies focuses on patient perception of their own pain and function.

Regarding limitations, the studies included used highly variable sample sizes, and some did not even calculate the sample size. The heterogeneity of muscle development exercise programmes did not allow to compare them globally due to the different doses of the intervention and different methodology of the exercises applied. Even so, the authors of this study attempted to complement the systematic review with quantitative analysis by group of studies. However, only two or three studies out of eight could be compared in each of the seven individual meta-analyses conducted regarding pain, strength (ABD, IR, ER), ROM in shoulder flexion and disability. The lack of homogeneity of the interventions implied insufficient data to generate general conclusions. Additionally, with such a small number of compared studies, one of them had a weight over 90%, which proved the decisive influence the results. The above factors resulted in poor and inconclusive results in favour of eccentric training. Therefore, the inclusion of quantitative analyses in this paper was discarded. This limitation leads us to prospectively consider the meta-analysis of future homogeneous interventions that may be published.

The authors also propose studies that apply exclusively homogeneous exercise programmes and parameters, i.e., without combining them with other therapies to assess their real effectiveness. Therefore, the systematic reviews could be also complemented with comprehensive meta-analyses. Given the response of the tendinopathies to the load, scientific evidence should be generated on how this load is evaluated and managed in RC tendinopathy patients.

After analysing and comparing different active physical exercise programmes in *good* methodological quality studies, this review, similar to many other studies [17,18,19,20], argues that these programmes could be applied to improve pain and function in RC tendinopathy patients, without highlighting the efficacy of one over the other. Also worth mentioning is the assessment of patients perception of the improvement achieved with the therapies.

## 5. Conclusions

All the physical exercise programmes based exclusively on muscle development covered by this systematic review were effective in the treatment of rotator cuff tendinopathy with the aim of improving shoulder pain and function. However, no solid results were obtained when the different interventions were compared due to their heterogeneity. Only one study found statistically and clinically significant differences in favour of eccentric training compared to a conventional exercise programme, i.e., global shoulder exercises, assessed using the Western Ontario Rotator Cuff index questionnaire.

The review considered exercise programmes based on isolated eccentric contractions, combinations of concentric and eccentric contractions, and isolated concentric contractions, ordered from most to least frequent. The exercises consisted of: open and closed kinetic chain exercise programmes, activities with and without coactivation of the glenohumeral muscles, global shoulder exercises, exercises with high eccentric loads, and supervised activities in consultation or at home without supervision. The interventions lasted 4, 6, 8 and 12 weeks, with follow-up up to week 26 from the start of the intervention.

Tools used to measure patients’ perception featured far more than other, more objective measuring instruments such as dynamometers, inclinometers or goniometers. The most widely used Patients Related Outcome Measures were the Numeric Rating Scale and Visual Analogue Scale for pain, as well as Shoulder Pain and Disabilities Index and Western Ontario Rotator Cuff index for pain and function. As a clinical contribution, we would like to highlight the benefits of actively involving the user in physiotherapy to ensure greater adherence to treatment.

Finally, based on the referenced literature, it is recommended that attention be focused on the appropriate amount of load to be applied, rather than on the method of physical exercise used.

## Figures and Tables

**Table 1:** Characteristics of studies.

Characteristics of Studies			
Study/Objective	Characteristics of Participants	Intervention/Comparison	Outcome Measures/Results
Maenhout, A. et al., 2013 [33] Study objective: To investigate superior value of adding heavy load eccentric training to conservative treatment in patients with subacromial impingement. Level of evidence: Therapy, 2b (OCEBM)	*n* = 61 Sex: 25 men/36 women Age (Mean ± SD): Group 1: 40.2 ± 12.9 years Group 2: 39.4 ± 13.1 years Inclusion criteria: >18 years; unilateral pain ≥3 months in the anterolateral region of the shoulder; painful arc; 2 out of 3 impingement tests positive (Hawkins, Jobe and/or Neer tests), 2 out of 4 resistance tests painful and pain with palpation of the supraspinatus and/or infraspinatus tendon insertion. Exclusion criteria: partial/full RC ruptures; shoulder surgery, fracture, dislocation; traumatic onset of the pain; osteoarthritis; frozen shoulder, traumatic glenohumeral instability; shoulder nerve injuries; concomitant disorders (cervical pathology or systemic musculoskeletal disease); physiotherapy and/or corticosteroids within 2 months of the intervention.	Heavy-load eccentric exercises Both groups: 1 physiotherapy session/week for the first 6 weeks and 1 physiotherapy session/2 weeks for the last 6 weeks. Duration: 12 weeks. Group 1—Traditional RC strength training + heavy load eccentric training (*n* = 31): RC exercises + heavy load eccentric exercise (ABD in scapular plane with thumb up). Frequency and parameters: 2 times/day—3 × 15 rep. Group 2—Traditional RC strength training (*n* = 30): RC exercises (IR and ER). Frequency and parameters: 1 time/day—3 × 10 rep.	Variables evaluation at 6 and 12 weeks. Main variables evaluated with: - SPADI In both groups, pain and function, as measured by the SPADI score, improved significantly over time (*p* < 0.001). There were no significant differences between groups. - Dynamometer (isometric strength of ABD in the scapular plane at 0°, 45° and 90°, ER and IR). Both groups showed a significant improvement in isometric strength of ABD in the scapular plane at 0° (*p* < 0.001) and 45° (*p* < 0.001), ER (*p* < 0.001) and IR (*p* = 0.038).
Blume, C. et al., 2015 [34] Study objective: To compare the effectiveness of an eccentric progressive resistance exercise intervention to a concentric progressive resistance exercise intervention in adults with subacromial impingement syndrome. Level of evidence: Therapy, 1b (OCEBM)	*n*= 34 Sex: 14 men/20 women Age (mean ± SD): 49.4 ± 15.6 years Inclusion criteria: ≥18 years; at least 1 of 3 positive tests (Neer, Hawkins-Kennedy and coracoid ADD impingement tests); 1 negative RC tear test. Exclusion criteria: shoulder, cervical, or thoracic surgeries; shoulder dislocation; fracture; labral tear; full-thickness RC tear; adhesive capsulitis; rheumatic disease; pregnancy or medical condition that precluded them from performing resisted exercises.	Eccentric vs. concentric exercises Both groups: Exercise parameters: 3 × 12 rep. of each exercise at 70% MR. Exercises: Seated full can, sidelying IR, sidelying ER, supine protraction, sidelying horizontal ABD, sidelying ABD and prone shoulder extension in neutral rotation. Home exercise programme: pectoralis minor and posterior shoulder stretching, thoracic spine self-mobilization into extension, and pain-free flexion AROM and ABD standing in front of mirror to monitor for excessive scapular elevation. Frequency and duration: 2/week during 8 weeks. Group 1—Concentric progressive resistance exercises (*n* = 18): Lifting portion of the exercises with the therapist repositioning the weight to the start position to avoid resistance in the lowering (eccentric) portion of the exercise. Group 2—Eccentric progressive resistance exercises (*n* = 16): Lowering portion of the exercises with the therapist repositioning the weight to the starting position to avoid resistance during the lifting (concentric) portion of each exercise.	Valiables evaluation at 5 and 8 weeks. Variables evaluated with (does not specify which is the primary variable): - DASH - Inclinometer (shoulder AROM of ABD in the scapular plane) - Dynamometer (for isometric strength) No significant differences were found in DASH (*p* = 0.890), shoulder ABD (*p* = 0.373), ABD torque (*p* = 0.421) and ER torque (*p* = 0.933) between groups. Significant improvements were found for all variables up to week 5 (*p* < 0.05) regardless of group assignment. All measures except arm elevation ROM (*p* = 0.302) continued to show significant improvement from week 5 to week 8 (*p* < 0.05).
Granviken, F. et al., 2013 [35] Study objective: To compare the different effects of home exercise and supervised exercise on pain and disability for patients with subacromial impingement. Level of evidence: Therapy, 1b (OCEBM)	*n* = 46 Sex: 24 men/22 women Age (mean ± SD): Group 1: 48.2 (9.8) years Group 2: 47.6 (10.0) years Inclusion criteria: Patients aged between 18–65 years; unilateral shoulder pain > 12 weeks; 3 positive diagnostic clinical tests (painful arc between 60 and 120^°^ during active ABD; infraspinatus and Hawkins-Kennedy test.) Exclusion criteria: Glenohumeral instability; acromioclavicular joint pathology; labrum pathology on imaging; full-thickness RC tears; glenohumeral osteoarthritis; undergone shoulder surgery; insufficient language capability; cervical spine problems; rheumatoid arthritis;other physical or serious mental illness.	Home vs. supervised exercise Both groups: - Education in shoulder injuries (anatomy and RHB processes) - Scapular stabilization, RC and mobility exercises without pain. - Stretching exercises. - Training diary. Frequency and duration: 2 times/day for 6 weeks. Parameters: 3 × 30 rep. Group 1—Home exercises (*n* = 23): 1 supervised treatment + home exercises. Group 2—Supervised exercises (*n* = 23):10 supervised treatments + home exercises.	Variables evaluated at 6 and 26 weeks Main variable evaluated with: - SPADI Secondary variables evaluated with: - NPRS - Clinic test (painful arc, infraspinatus and Hawkins-Kennedy test) - FABQ - Digital inclinometer (shoulder AROM in flexion, ABD, ER and IR) - Scale to measure self-reported work status - Scale to measure participant satisfaction There was no statistically significant difference between home and supervised exercises in SPADI at 6 weeks (MD 0 points; 95% CI (−14, 14) and 26 weeks (MD −2 points; 95% CI (−21, 17) of follow-up. There were no significant differences between groups in pain, physical activity, or work on the FABQ and ROM. No participant reported full recovery in terms of perceived benefit. One subject was dissatisfied with treatment.
Chaconas, E. J. et al., 2017 [36] Study objective: To compare outcomes, for individuals diagnosed with subacromial pain syndrome, performing a 6-week protocol of eccentric training of the shoulder external rotators compared to a general exercise protocol. Level of evidence: Therapy, 2b (OCEBM)	*n* = 48 Sex: 28 men/20 women Age (mean ± SD): 46.8 ± 17.29 years Inclusion criteria: ≥3 positives tests of: Neer, Hawkins-Kennedy and the empty can test; pain with resisted ER; palpable tenderness at the insertion of the supraspinatus or infraspinatus; painful arc between 60–120° active ABD; shoulder pain ≥3 months duration Exclusion criteria: Red flags; full thickness supraspinatus or infraspinatus tendon; adhesive capsulitis and history of shoulder surgery.	Eccentric vs. general shoulder exercises: Both groups: Diary exercise. Scapular retraction: - Frequency: 1 time per day. - Parameters: 2 × 10 rep. Posterior shoulder stretching: - Frequency: 1 time per day. - Parameters: 3 × 30–45 seg. Duration: 6 weeks. Group 1—Eccentric exercises (*n* = 25): Eccentric exercises with scapular retraction + posterior shoulder stretching. Eccentric exercises of external rotators: - Frequency: 1 time per day. - Parameters: 3 × 15 rep. Group 2—General exercises (*n* = 23): Shoulder general exercises protocol (flexion, ABD, active scapular retraction and posterior shoulder stretching). Active ROM: - Frequency: 1 time per day. - Parameters: 2 × 10 rep.	Variables evaluated at 3, 6 weeks and 6 months. Main variables evaluated with: WORC NPRS Secondary variables evaluated with: Dynamometer (isometric strength of shoulder in ABD, ER and IR) UQYBT GROC After 3 weeks only NPRS (*p* < 0.03) and the isometric strength in ER (*p* < 0.001) showed a statistically significant interaction effect. At the end of the treatment (6th week), a significant interaction was identified for the mean and worst values of NPRS (*p* < 0.001), the ER strength ( < 0.001), and the proportions of external rotator to abductor and external rotator to internal rotator strength (*p* < 0.04). After 6 months, secondary outcomes improved for pain on average and pain on worst (*p* < 0.02), ABD, ER and IR strength (*p* < 0.02). Secondary outcomes of GROC, AROM, UQYBT and strength ratios were not statistically significant in the multilevel model after 6 months.
Heron, S. et al., 2017 [23] Study objective: To assess the efficacy of three different exercise programmes in treating RC tendinopathy/shoulder impingement syndrome. Level of evidence: Therapy, 1b (OCEBM)	*n* = 120 Sex: 71 men/49 women Age (mean): 49, 9 years Inclusion criteria: shoulder pain for ≥3 months; no passive limitation of ROM suggestive of adhesive capsulitis; pain on isometric RC testing; pain on Hawkins–Kennedy or empty can tests; able to read and write English. Exclusion criteria: symptoms of cervical radiculopathy; diagnosed inflammatory disorder; neurological disorder; widespread pain condition; complete RC tear (positive drop arm test and/or Oxford scale grade II and/or less strength of the RC); history of shoulder sugery.	Open kinetic chain vs. closed kinetic chain vs. mobility exercises All groups: Anterior and posterior shoulder stretching (5 rep/2 times per day). Frequency and duration: 2 times/day during 6 weeks. Parameters: 3 × 10 rep. Group 1—Open kinetic chain exercises (*n* = 40): ER, IR and ABD exercises with a resistance elastic band. Group 2—Closed kinetic chain exercises (*n* = 40): Double-arm wall press up, quadrupedal press up and a seated position and pressed their hands into the chair, as if trying to lift their body. Group 3—Mobility exercises) (*n* = 40): IR, ER and ABD exercises from self-passive mobility to free active mobility.	Variables evaluated at 6 weeks. Main variable evaluated with: - SPADI All groups showed a significant reduction in SPADI over the 6 week follow-up. Change of intra-group mean in SPADI; (Effect size). Group 1: 12; *p* = 0.0001 (0.56); Group 2: 9; *p* = 0.0002 (0.63); Group 3: 9; *p* = 0.0002 (0.49). There were no significant differences between groups. (Kruskal-Wallis test): Change in SPADI mean (95% CI): Group 1: −3.5 (−5, 12); Group 2: −0.5 (−3, 15); Group 3: −4.0 (−5, 17). There were no clinically significant differences in SPADI during follow-up.
Dejaco, B. et al., 2017 [24] Study objective: To investigate the effectiveness of isolated eccentric versus conventional exercise therapy in patients with RC tendinopathy. Level of evidence: Therapy, 1b (OCEBM)	*n*= 36 Sex= 19 men/17 women Age (mean ± SD) Group 1 = 50.2 ± 10.8 years Group 2 = 48.6 ± 12.3 years Inclusion criteria: 18–65 years; both genders; unilateral subacromial pain ≥3 months; 2 out of 3 positive impingement tests (empty-can, Hawkins–Kennedy and modified Neer test). Exclusion criteria: Subjective feeling of instability and positive apprehension sign; positive scapular assistance and/or resistance test; partial/full ruptures of RC; calcifications >4 mma; acromion type III (according to Bigliani criteria); bursitis; history of shoulder fracture and/or shoulder surgery; cervical radiculopathy; adhesive capsulitis; systemic diseases; corticosteroid injection 3 months prior to inclusion.	Eccentric vs. conventional exercises Both groups: 1 session of physiotherapy per week (during the first 6 weeks) and 3 sessions per week (during the last 6 weeks) Frequency and duration: Diary home exercises during 12 weeks. Parameters: 3 × 8 rep. (First increased in rep. to 15 and then in load). Stretching: Minor pectoral and cross ADD for posterior shoulder musculature and capsule structures. Group 1—Eccentric exercises (*n* = 20): 2 differents exercises 2 times/day. - ER in supine with shoulder 90° ABD. ABD in scapular plane until 90° (eccentric phase). Group 2—Conventional exercises (*n* = 16): 8 differents exercises 1 time/day - ABD “full can” in scapular plane until 90°. - IR and ER at 0° ABD. - Shoulder shrugs. - Knee push-up. - Horizontal ABD in prone position with ER.	Variables evaluated at 6, 12 and 26 weeksMain variable evaluated with: - CM The score increased significantly in both groups (0–26 weeks). Group 1: 14.4 points (*p* < 0.001) and Group 2: 9.8 points (*p* < 0.001). There were no significant differences between groups (4.6 points). - VAS The score improved significantly in both groups. Group 1: −19.9 mm (*p* < 0.001) and Group 2: −22.3 mm (*p* < 0.001). There were no significant differences between groups (2.4 mm). Secondary variables evaluated with: - Goniometer (shoulder ROM in flexion-elevation, ABD-elevation and ER) - Dynamometer (isometric strength of shoulder ABD) Each group obtained a slight improvement in ROM and isometric strength at 26 weeks. No statistically significant differences were found either intra- or inter-group.
Vallés-Carrascosa, E. et al., 2018 [22] Study objective: To compare the effect on pain, active ROM and shoulder function of an exercise protocol performed with pain <40 mm Visual Analogue Scale and without pain, in patients with subacromial syndrome. Level of evidence: Therapy, 2b (OCEBM)	*n* = 22 Sex: 10 men/12 women Age (mean (1st quartile; 3rd quartile)) Group 1: 60.0 (47.0; 70.0) years Group 2: 57.0 (49.0; 70.0) years Inclusion criteria: 25–70 years; referred to rehabilitation services after a diagnosis of subacromial syndrome; painful arc upon active lifting of the upper limb (ABD: 60°–120°) Exclusion criteria: RC tears; shoulder surgery in the last 3 months; frozen shoulder; shoulder prosthesis; fibromyalgia; malignant neoplasm; history of rheumatic or chronic inflammatory disease.	With vs. withouth pain Both groups: RC exercises (in the affected limb), scapular stabilization exercises (both limb) and upper limb stretching (both limbs). Frequency and duration: 5 times/week during 4 weeks. (The stretchings were performed 3 times/session). Parameters: 3 × 10 rep (rest twice the duration of a series). Group 1—With pain (*n* = 11): Exercises with pain (no >40 mm on the VAS scale). Group 2—Without pain (*n* = 11): Exercises without pain (0 mm on VAS scale).	Variables evaluated at 4 weeks Main variables evaluated with: VAS Goniometer (shoulder AROM in flexion, extension, ABD, ADD, ER and IR) Secondary variable evaluated with: CM Both groups improved significantly. VAS and CM improved significantly (*p* < 0.01), as did AROM (*p* < 0.05). There were no significant differences between groups (*p* > 0.05).
Bourdreau, N. et al., 2019 [37] Study objective: To compare the short-term efficacy of adding glenohumeral adductor coactivation to a RC strengthening program to improve function, reduce symptoms and increase acromiohumeral distance in adults with RC tendinopathy. Level of evidence: Therapy, 1b (OCEBM)	*n* = 42 Sex: 20 men/22 women Age (mean ± SD): Group 1: 49.6 ± 13.2 years Group 2: 50.2 ± 10.9 years Inclusion criteria: 18–65 years; symptoms lasting >1month; a painful arc in active flexion or ABD; positive Neer or Hawkin’s Kennedy test; pain when resisting humeral ER or ABD; English or French languages. Exclusion criteria: Full-thickness RC tear; shoulder surgery; shoulder capsulitis, osteoarthritis or traumatic instability; rheumatoid arthritis; systemic inflammatory or neurologic condition; corticosteroid injections in the affected shoulder within the past 6 weeks.	Exercises with co-activation of glenohumeral musculature vs. without it Both groups: Serratus anterior, trapezius and glenohumeral muscles (ER and IR) strengthening exercises. Parameters: 3 × 10 rep. Frequency and duration: 1 time/day—7 days/week—during 6 weeks. Group 1—Co-activation of glenohumeral muscles (*n* = 21): Glenohumeral muscles exercises with recruitment of minor pectoral and latissimus dorsi, with visual feedback (EMG). The rest of exercises were done in the same way. Group 2—Withouth co-activation of glenohumeral muscles (*n* = 21): Glenohumeral muscles exercises without recruitment of minor pectoral and latissimus dorsi, with visual feedback (EMG). The rest of exercises were done in the same way.	Variables evaluated at 3 and 6 weeks Main variable evaluated with: - DASH Secondary variables evaluated with: - WORC - VAS - Ultrasound (to AHD at 0°, 30° and 60° ABD) No statistically significant differences were obtained between groups for DASH (*p* = 0.522), WORC (*p* = 0.421), VAS (*p* = 0.140) and AHD (*p* > 0.055). Significant time effects were obtained for WORC and VAS (*p* < 0.001).

Abbreviations: ABD, abduction; ADD, adduction; AHD, acromio-humeral distance; AROM, active range of motion; CM, Constant Murlay; DASH, Disabilities of Arm, Shoulder and Hand; EMG, electromyography; ER, external rotation; FABQ, Fear Avoidance Beliefs Questionnaire; GROC, global rating of change; IR, internal rotation; MD, mean difference; MR, maximum repetition; NPRS, Numeric Pain Rating Scale; OCEBM, Oxford Centre for Evidence-Based Medicine; OSI, Ocupational Stress Indicator; RC, rotator cuff; RHB, rehabilitation; ROM, range of motion; SD, standard desviation; SPADI, Shoulder Pain And Disabilities Index; UQYBT, Upper Quarter Y-Balance test; VAS, Visual Analogue Scale; WORC, Western Ontario Rotator Cuff Index.

**Table 2:** Assessment of the methodological quality of the studies using PEDro scale.

Study	Criteria											Total
	1	2	3	4	5	6	7	8	9	10	11	
Maenhout, A. et al. [33]	√	√	*X*	√	*X*	*X*	*X*	√	√	√	√	6
Blume, C. et al. [34]	√	√	√	√	*X*	*X*	√	√	√	√	√	8
Granvinken, F. et al. [35]	√	√	√	√	*X*	*X*	√	√	√	√	√	8
Chaconas, E. J. et al. [36]	*X*	√	*X*	√	*X*	*X*	√	√	*X*	√	√	6
Heron, S. et al. [23]	√	√	√	√	*X*	*X*	√	*X*	√	√	√	7
Dejaco, B. et al. [24]	√	√	√	√	*X*	*X*	*X*	√	√	√	√	7
Vallés-Carracosa, E. et al. [22]	√	√	√	√	*X*	*X*	*X*	√	√	√	√	7
Bourdreau N. et al. [37]	√	√	√	√	*X*	*X*	√	√	√	√	√	8

Data extracted from PEDro database. Criteria: 1, Eligibility criteria were specified (not used for score); 2, Subjects were randomly allocated to groups; 3, Allocation was concealed; 4, Groups were similar at baseline regarding the most important prognostic indicators; 5, There was blinding of all subjects; 6, There was blinding of all therapists who administered the therapy; 7, There was blinding of all assessors who measured at least one key outcome; 8, Measures of at least one key outcome were obtained from more than 85% of the subjects initially allocated to groups; 9, All subjects for whom outcome measures were available received the treatment or control condition as allocated or, where this was not the case, data for at least one key outcome was analysed by ‘intention-to-treat’; 10, The results of between-group statistical comparisons were reported for at least one key outcome; 11, The study provides both point measures and measures of variability for at least one key outcome). √ criteria met; *X:* criteria not met.

**Table 3:** Assessment procedures used in the selected studies.

	Assessment Tests					
Studies	Hawkins-Kennedy Test	Neer Test	Isometric Test	Painful Arc	Empty Can Test	Others *
Maenhout, A. et al. [33]	√	√	√	√		√
Blume, C. et al. [34]	√	√				√
Granvinken, F. et al. [35]	√		√	√		√
Chaconas, E. J. et al. [36]	√	√	√	√	√	√
Heron, S. et al. [23]	√		√		√	
Dejaco, B. et al. [24]	√	√			√	
Vallés-Carracosa, E. et al. [22]				√		
Bourdreau N. et al. [37]	√	√	√	√		
**Total**	7	5	5	5	3	4

* Coracoid extraction, sensitivity in tendon insertion of the supraspinatus or infraspinatus, Jobe’s test, infraspinatus test.

**Table 4:** Grouping of studies according to muscle contraction mode.

Muscle Contraction Types	Number of Studies
Concentric + Eccentric (The load is applied during the concentric and eccentric phase)	4 [22,23,35,37]
Concentric (The load is only applied during the concentric phase)	1 [34]
Eccentric (The load is only applied during the eccentric phase)	4 [24,33,34,36]

**Table 5:** Duration and monitoring of the intervention.

**STUDIES**	**Monitoring and Evaluation of Variables**	**WEEKS**							
		**0**	**3**	**4**	**5**	**6**	**8**	**12**	**26**
	**Maenhout, A. et al.** [33]	√				√		√	
	**Blume, C. et al.** [34]	√			√		√		
	**Granvinken, F. et al.** [35]	√				√			√
	**Chaconas, E. J. et al.** [36]	√	√			√			√
	**Heron, S. et al.** [23]	√				√			
	**Dejaco, B. et al.** [24]	√				√		√	√
	**Vallés-Carrascosa, E. et al.** [22]	√		√					
	**Bourdreau, N. et al.** [37]	√	√			√			

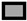
 End of intervention; √: Time of evaluation of the variables.

**Table 6:** PROMs used in selected studies.

	Patient Reported Outcome Measures							
Studies	NPRS/VAS	SPADI	WORC	DASH	CM	FABQ	UQYBT	GROC
Maenhout, A. et al. [33]		√						
Blume, C. et al. [34]				√				
Granvinken, F. et al. [35]	√	√				√		
Chaconas, E. J. et al. [36]	√		√				√	√
Heron, S. et al. [23]		√						
Dejaco, B. et al. [24]	√				√			
Vallés-Carracosa, E. et al. [22]	√				√			
Bourdreau N. et al. [37]	√		√	√				
**Total**	5	3	2	2	2	1	1	1

Abbreviations: CM, Constant Murlay Score; DASH, Disabilities of Arm, Shoulder and Hand; FABQ, Fear Avoidance Beliefs Questionnaire; GROC, Global Rating of Change; NPRS, Numeric Pain Rate Scale; PROMs, Patients Related Outcome Measures; SPADI, Shoulder Pain and Disability Index; UQYBT, Upper Quarter Y-Balance test; VAS, Visual Analogue Scale; WORC, Western Ontario Rotator Cuff Index.

**Table 7:** Interventions based on muscle development with load and their effectiveness based on the variables studied.

	ROM	ISOMETRIC STRENGHT	SPADI	WORC	DASH	NPRS/VAS	CM	FABQ	UQYBT	GROC
Maenhout, A. et al. [33]		√	√							
Blume, C. et al. [34]	√	√			√					
Granvinken, F. et al. [35]	√		√			√		√		
Chaconas, E. J. et al. [36]	√	√		√		√			√	√
Heron, S. et al. [23]			√							
Dejaco, B. et al. [24]	√	√				√	√			
Vallés-Carrascosa, E. et al. [22]	√					√	√			
Bourdreau, N. et al. [37]				√	√	√				

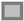
 Significant and effective; 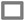 Not significant. √ Variable measured in study; Abbreviations: CM Constant Murlay; DASH, Disabilities of Arm, Shoulder and Hand; FABQ, Fear Avoidance Beliefs Questionnaire; GROC, Global Rating of Change; NPRS, Numeric Pain Rating Scale; OSI, Ocupational Stress Indicator; ROM, range of motion; SPADI, Shoulder Pain and Disabilities Index; UQYBT, Upper Quarter Y-Balance test; VAS, Visual Ananolgue Scale; WORC, Western Ontario Rotator Cuff Index.

## Abbreviations

ABD	Abduction
AHD	Acromio-Humeral Distance
AROM	Active Range of Motion
CI	Confidence Interval
CM	Constant Murlay
CROB	Cochrane Risk of Bias
DASH	Disabilities of Arm, Shoulder and Hand
EMG	Electromyography
ER	External Rotation
FABQ	Fear Avoidance Beliefs Questionnaire
GROC	Global Rating of Change
IR	Internal Rotation
MeSH	Medical Subject Headings
MR	Maximum Repetition
MD	Mean Difference
MDC	Minimum Detectable Change
MDP	Muscle Development Program
NPRS	Numeric Pain Rating Scale
OCEBM	Oxford Centre for Evidence-Based Medicine
OSI	Ocupational Stress Indicator
PROMs	Patient Related Outcome Measures
RC	Rotator Cuff
RCT	Randomized Controlled Trial
RHB	Rehabilitation
ROM	Range of Motion
SD	Standard Desviation
SE	Standard Error
SMD	Standarized Mean Difference
SPADI	Shoulder Pain and Disabilities Index
UQYBT	Upper Quarter Y-Balance test
VAS	Visual Analogue Scale
WORC	Western Ontario Rotator Cuff Index

## Appendix A

**Table A1:** Search strategy terms ordered by meaning.

MeSH Terms * and Others	Identifier
*tendinopathy* or tendonopathy or tendinosis or tendinoses or tendonosis or tendonoses or tendinitis or tendinitides or tendonitis or tendonitides or “shoulder tendinopathy” or “*shoulder impingement syndrome”* or “shoulder impingement” or “subacromial impingement syndrome” or “subacromial bursitis”	**1**
“*rotator cuff*” or supraspinatus or infraspinatus or subscapularis or “teres minor” or “long head of biceps”	**2**
*“isometric contraction*” or “*isotonic contraction*” or concentric* or eccentric * or exercise* or resistance* or load *	**3**
*“randomized controlled trial”*	**4**

* MeSH terms are in italics.

## Appendix B

**Table A2:** Search strategy in the different databases.

Database	Search Strategy	Identifier
**Pubmed**	(tendin * or tendon * or “shoulder tendinopathy” or “shoulder impingement *” or “shoulder burs *” or “subacromial impingement” or “subacromial impingement *” or “subacromial bursitis”) and (“rotator cuff” or supraspinatus or infraspinatus or subscapularis or “teres minor” or “long head of bíceps”) and (isometric * or isotonic * or concentric* or eccentric * or exercise * or “resistance training” or load *) Filter: “clinical trial”	1, 2 and 3
**WOS**	(tendin * or tendon * or “shoulder tendinopathy” or “shoulder impingement *” or “shoulder burs*” or “subacromial impingement” or “subacromial impingement*” or “subacromial bursitis”) and (“rotator cuff” or supraspinatus or infraspinatus or subscapularis or “teres minor” or “long head of biceps”) and (isometric * or isotonic * or concentric* or eccentric * or exercise * or resistance * or load *) and random * Filter: “article”	1, 2, 3 and 4
**PEDro**	“rotator cuff tend *” and “strength training” and pain and “upper arm, shoulder or shoulder girdle” and musculoskeletal and “clinical trial”	1 and 2 *
**Cinahl**	(tendin * or tendon * or “shoulder tendinopathy” or “shoulder impingement *” or “shoulder burs*” or “subacromial impingement” or “subacromial impingement*” or “subacromial bursitis”) and (“rotator cuff” or supraspinatus or infraspinatus or subscapularis or “teres minor” or “long head of bíceps”) and (isometric * or isotonic * or concentric* or eccentric * or exercise * or “resistance training” or load *) Filter: “clinical trial”	1, 2 and 3
**Scopus**	(tendin * or tendon * or “shoulder tendinopathy” or “shoulder impingement *” or “shoulder burs*” or “subacromial impingement” or “subacromial impingement*” or “subacromial bursitis”) and (“rotator cuff” or supraspinatus or infraspinatus or subscapularis or “teres minor” or “long head of biceps”) and (isometric* or isotonic * or concentric* or eccentric* or exercise * or “resistance training” or load *) and random * Filter: “article”	1, 2, 3 and 4
**Dialnet ****	(tendin * or tendon * or “shoulder tendinopathy” or “shoulder impingement *” or “shoulder burs *” or “subacromial impingement” or “subacromial impingement*” or “subacromial bursitis”) AND (isometric * or isotonic * or concentric * or eccentric * or exercise * or “resistance training” or load *) and random * (“rotator cuff” or supraspinatus or infraspinatus or subscapularis or “teres minor” or “long head of biceps”) and (isometric * or isotonic * or concentric * or eccentric * or exercise* or “resistance training” or load *) and random * Filter: “artículos de revista”	1,2,3 and 4

* Advanced search was performed on PEDro. The search strategy corresponds to the following sections: “therapy”, “problem”, “body part”, “subdiscipline” and “method” respectively. ** Two complementary search strategies were carried out due to the limitation of the number of terms in the search engine.
